# Healing wounded trees: clinicians’ perspectives on treatment of complex posttraumatic stress disorder

**DOI:** 10.3389/fpsyt.2024.1356862

**Published:** 2024-04-09

**Authors:** Boris Drožđek, Jan Rodenburg

**Affiliations:** De Hemisfeer, Den Bosch, Netherlands

**Keywords:** posttraumatic stress disorder, treatment, developmental psychology, ecological perspective, psychotherapy, complex PTSD (CPTSD)

## Abstract

While treatment guidelines agree on the first-line interventions for the treatment of posttraumatic stress disorder (PTSD), there is an ongoing debate between experts regarding the treatment of complex posttraumatic stress disorder (C-PTSD). As scientific research is slowly emerging, different treatment approaches are used in clinical practice This article aims to provide a set of treatment options for C-PTSD in adult survivors of repeated exposure to severe violence and abuse, both in childhood and later on in life. The developmental-contextual perspective on mental health forms the basis of this approach. This perspective is elaborated using the tree metaphor. Then, several treatment strategies are suggested. The presented strategies are a combination of the existing evidence-based approaches for the treatment of PTSD and personality disorders. They target psychological damage in survivors while taking their developmental trajectories and ecological environments into consideration. The treatment model presented is based on longstanding clinical practice and it may be a promising framework for treating C-PTSD. However, it still needs to be scientifically examined for acceptability and effectiveness.

## Introduction

PTSD is a diagnostic entity characterized by the core symptoms of re-experiencing, avoidance/numbing, and hyper-arousal/sense of threat. C-PTSD includes the same core symptoms in conjunction with the disturbances in self-regulatory capacities, like emotion regulation difficulties, disruptions in relational capabilities, alterations in attention and consciousness (e.g., dissociation), adversely affected belief systems, and somatic distress and disorganization (1). C-PTSD is assumed to occur after exposure to severe, prolonged, or repeated stressors, like in survivors of childhood neglect, physical and sexual abuse, domestic violence, sex trafficking, exposure to torture, genocide, or other forms of organized violence.

In the past two decades, we have witnessed a proliferation of meta-analytic studies of psychological treatments for PTSD. These studies show that cognitive behavior therapy (TF-CBT) and eye movement desensitization and reprocessing (EMDR) are efficacious in alleviating symptoms in the short term (2, 3). EMDR is suggested to be more efficient than TF-CBT in reducing PTSD symptoms and anxiety (4). Another recent study has found that prolonged exposure (PE) and cognitive processing therapy (CPT) are equally effective (5). According to international guidelines, TF-CBT and EMDR are the first-line treatment interventions (6). More recently, written exposure therapy and narrative exposure therapy (NET) have also been recommended. They were associated with a lower dropout risk, compared to TF-CBT and EMDR, but less efficacious (7).

On the other hand, the treatment of C-PTSD is still a subject of discussion. The guidelines (6) recommend the phase-oriented or sequenced approach (1). This approach starts with the stabilization phase to ensure survivors’ safety by reducing self-regulation problems and improving emotional, social, and psychological competencies. Then, the phase focusing on the processing of traumatic memories takes place, and trauma-focused interventions are used. As relapses are expected in the second phase, treatment may switch back and forth between the first and the second phase, aiming at re-consolidating skills before continuing with trauma processing. In the third, the reintegration phase, treatment gains are consolidated, and survivors are assisted with adapting to current life circumstances. This approach assumes that C-PTSD patients are not stable enough to tolerate strong emotions and that a trauma-focused intervention should be preceded by emotion regulation skills training. It is believed that survivors’ psychosocial and environmental resources should be strengthened first, as exposure to severe, prolonged, and repeated trauma deteriorates social, emotional, and psychological resources beyond the PTSD complaints. Therefore, survivors may experience difficulties in responding to and recovering from the next stressor, leading to a downward spiral of resource loss (8). According to the classical biomedical perspective, psychosocial conditions play an essential role in determining resilience to pathology (9). However, a body of research on psychosocial, resources-oriented interventions targeting the ecological environment of trauma populations is limited.

The other perspective (10) on C-PTSD treatment is unimodal. It argues that the stabilization phase is unnecessary and that C-PTSD treatment can start with first-line trauma-focused interventions right away, just like in the treatment of PTSD. It is believed that the sequenced approach delays or denies access to effective evidence-based trauma-focused treatment from which survivors might profit. Also, it may demoralize patients by inadvertently communicating that they are incapable of dealing with their traumatic memories, thereby reducing their self-confidence and motivation for trauma processing. Further, emotional self-regulation problems, survivors’ interpersonal challenges, and a negative self-concept are thought to improve after the trauma-focused treatment and they do not need to be targeted separately (11, 12).

More recently, it was suggested (13) that these two conflicting perspectives have more in common than previously thought and that clinicians should consider more blended practices in treating C-PTSD. The argument for the unimodal vs. the phase-based perspective becomes redundant since unimodal therapies usually require stabilization interventions beforehand and follow-up interventions afterward to increase their efficacy. Since strong evidence specific to the treatment of the C-PTSD population is still lacking, it was recently suggested to tailor the treatment according to patients’ needs and symptoms (14).

Regarding the treatment duration, there is enough evidence indicating that patients with complex mental disorders are unlikely to respond to short-term treatments (15). It is believed that patients who were exposed to a combination of repeated childhood traumas with additional traumatic experiences in adulthood will suffer from more pervasive impacts. They may need treatment approaches lasting longer and being different from those efficaciously applied in acute PTSD samples (16).

The sequenced approach is expected to last from 15 months to 2 years, with weekly sessions tapering off over time based on the patient’s status in the third phase. The duration of each phase and points of transitions between phases are determined by clinical judgment. Moreover, treatment of most severely impaired survivors may last for several years and, or may be required intermittently throughout their life course (1).

The unimodal approach, like the EMDR therapy, is suggested to last a much shorter time. A format of 8 days combining EMDR (8 sessions of 90 minutes), imaginal and *in vivo* exposure (8 sessions of 90 minutes) with physical activity, psychoeducation, and supportive interventions offered upon indication in between sessions, has been studied. It has sorted clinically meaningful effects (12) in most participants presenting with a variety of trauma histories and multiple comorbidities. However, the sustainability of both approaches in the long-term (longer than 6 to 12 months posttreatment) has been insufficiently examined.

The C-PTSD treatment strategy presented in this article is based on clinical experience only. We suggest a combination of the existing evidence-based approaches for the treatment of PTSD and personality disorders and plead for therapeutic pluralism, instead of therapeutic sectarianism. We also argue that understanding of, and intervening in the dynamic relationship between survivors and their ecological environments will improve efficacy of the trauma therapy.

## The tree metaphor

American psychoanalyst Karin Horney (17) stated that humans have an innate potential for growth. According to her, therapy does nothing but remove obstacles to growth and enable humans to mature and realize their potential.

We imagine the genetic potential of a human being as a tree seed. A seed grows well if it has an intact genetic code. Moreover, it should be planted in fertile soil with sufficient nutrients and water. It develops roots anchoring a future tree in soil and starts growing above ground into a sprout. Then, sunlight and unpolluted air become essential for its further development. To expand freely, a tree also needs sufficient space, both under and above ground. A tree trunk with branches and leaves emerges, but the roots of a strong and resilient tree are always larger than a crown. All the essential ingredients for tree growth mentioned, are an analogy for epigenetic factors in human development.

In a favorable ecological environment, a tree starts using its resources and capabilities to develop further. Tree leaves make food through the process of photosynthesis. They use energy from sunlight, water, and carbon dioxide to create glucose, essential for tree growth. A mature and healthy tree is genuinely rooted in and intertwined with its ecosystem. Through photosynthesis, leaves produce not only food for a tree but also oxygen necessary for the survival of other species.

A tree also evolves various mechanisms to cope with environmental stressors. It may develop wider root systems to access enough water and nutrients and become more stable against strong winds. It may shed leaves during periods of extreme heat to reduce water loss through transpiration, or enter a state of dormancy during winter and conserve energy until conditions improve. This way, a tree can live for many years and survive in challenging environments, unless a powerful tornado or a flash of lightning strikes it.

A tree does not grow fast. It needs time to mature and become robust enough to respond to environmental stressors. At times, a young tree trunk needs to be assisted in development. Scaffolding should be built around it to prevent it from inclining and help it grow upright.

If a tree, whether a young or an old one, is moved from one place to another, to a different ecological context, it may suffer, dry up, and die. Or it just may be shaken and tormented, but survive. Sometimes, a tree will become stronger and grow better in changed and improved conditions. Migratory processes are always a challenge, both for trees and for humans.

**Figure 1:**
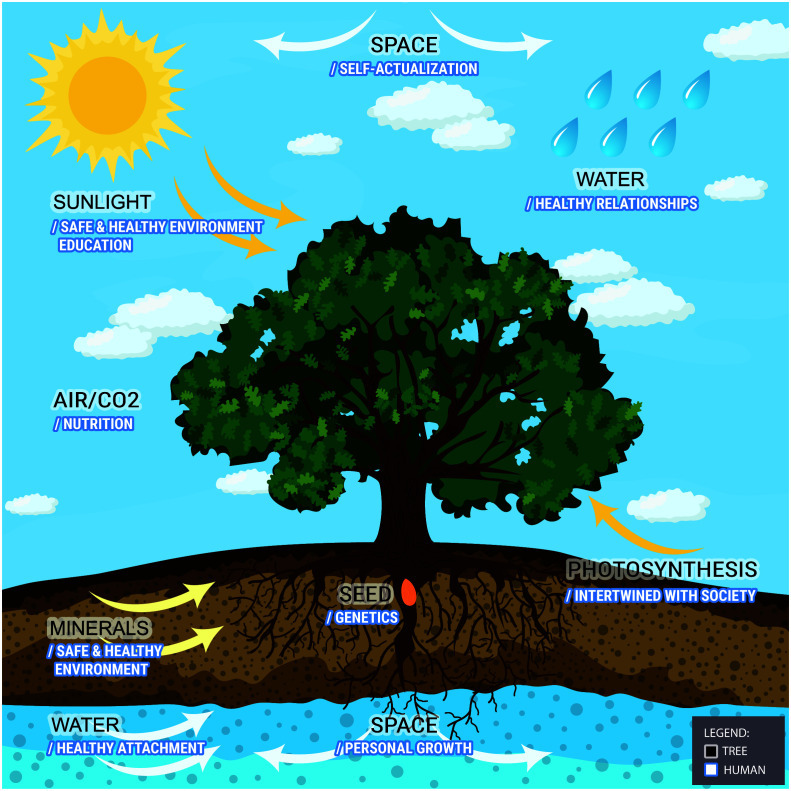
The tree metaphor.

We have coined this metaphor believing that it appropriately mirrors the relationship between an individual and context, its ecological surroundings. This relationship is bidirectional, nonlinear, and fluctuates throughout time. Like trees, humans often start suffering from psychopathology upon cumulation of potentially traumatic experiences over time, when their resilience capacities are worn out, and their environmental resources are weakened. They will recover only upon re-establishing a balance between sources of resilience and stress within their ecological environment (18) (**Figure 1**).

## What can go wrong with a tree and with a human?

Imagine that a seed has a damaged genetic code and does not receive proper instructions for growth, like a child whose mother has been exposed to toxic stress during pregnancy. Or that a seed is planted in dry and unfertile soil or does not have enough space for growth, like a child being brought up neglected in an affect-poor environment, restricted in a curious exploration of the world, harshly punished, not being rewarded, seen, and well taken care of. Or that soil is polluted with toxins, like in a child confronted with maltreatment and violence, transgenerational trauma, and an unsafe and unpredictable environment, or having parents with inadequate emotional self-regulatory capacities or severe sickness. Environmental stress will impair both a seed’s and a child’s developmental capacities.

Picture this: a seed is planted in soil with too much water or being extensively fertilized, like a child who is overprotected by caregivers and lacks “positive frustrations” necessary for the stimulation of psychological growth. Such a child may seem unharmed in the short term, but it may be confronted with severe challenges later in life.

When a seed develops into a sprout, it enters a “new world” above ground to which it becomes related and dependent. Its ecological environment becomes more complex and challenging as it needs to grow further in the soil and develop solid roots, as above it to produce a trunk, branches, and a lovely crown with many leaves. To start with, the growth process may be compromised by a lack of space, like a young individual who lacks caregivers’ acknowledgment of individual potential, demands, and character. Moreover, a young tree may not have enough sunshine, rain, and unpolluted air to develop further. An individual may lack a safe environment to build satisfactory relationships with others than caregivers and learn to manage life outside of a family home. It may also not receive good nutrition and education. Moreover, while a tree may be attacked by insects, pests, or bacteria a human may be exposed to major catastrophes, like wars, ecological disasters, hunger, poverty, or forced migrations.

The process of photosynthesis may also be compromised. Think of a child developing into an adult and facing obstacles in rounding up education and starting to work, building a family, getting firmly rooted in (sub)culture, developing interests in religion and spirituality, becoming socially and politically engaged, and contributing to society.

Returning to Horney’s (17) statement, one should first understand which obstacles individuals have faced throughout their psychosocial development, how they were impacted, and how they have coped with them. We consider the developmental perspective (19) to be crucial for understanding the underlying dynamics of the occurrence of mental health problems in humans in general, and in survivors of repeated trauma in particular.

## How to heal a wounded tree?: the developmental-contextual treatment approach

The following suggestions are based on the authors’ 30 years of professional experience with assisting adult survivors of severe and repeated trauma with diverse cultural backgrounds in an outpatient setting specialized in C-PTSD treatment. More specifically, our patients are asylum seekers and refugees who were submitted to war and, or torture in their countries of origin and have been forced to migrate after that, war veterans from different army forces who have been deployed in war zones throughout the world, survivors of childhood neglect, violence and abuse, and individuals being exposed to a combination of these stressful and traumatic experiences throughout their life courses. Most patients have previously been unsuccessfully treated for C-PTSD and comorbidity in other treatment settings, and are considered to be severely impaired.

## Developmental history: examining soil, water, sunshine, air, and space

A wounded or sick tree presents with a range of symptoms. It may lose half of its leaves, leaves may become deformed or get odd colors and dark spots. A tree’s structure may become unstable, its trunk and branches may dry up and die, and it may be attacked by insects. To treat a sick tree one should first understand the cause of the symptoms present, and determine whether a tree is sick because of insects, pests, bacteria, environmental stress, human activity, or a combination of different causes.

We argue that understanding psychopathology in humans goes beyond classifications of manifest diseases and checking for symptoms to establish diagnosis. Searching for diagnosis without understanding the logic of processes causing psychopathology will lead to category fallacy. Once a diagnosis is established, clinicians may tend to selectively overlook aspects of a patient that do not fit into a particular diagnosis and excessively focus on subtle features that appear to confirm an initial diagnosis (20). Moreover, diagnosis may limit vision and diminish the clinician’s ability to relate to a patient as a person. Understanding is, thus, more than taking a snapshot of the current situation.

Assisting adult trauma survivors and in particular, those with traumatic experiences in childhood, calls for a historical approach. At first, the clinician should take an in-depth personal developmental-contextual history (anamnesis) of a survivor. With this, it is vital to collect information about relevant events throughout life, from birth and the early years, followed by childhood, adolescence, and adulthood. When a survivor is a migrant, attention should also be paid to an exploration of pre-migration, migration, and post-migration periods in life. It is essential to collect both information about potentially traumatic life events and the development of resilience potentials on all levels of the survivor’s ecological environment, the micro, meso, exo, and macro levels (21).

One should question why an individual starts suffering from mental health problems at a particular time in life and what has prevented the manifestation of a disorder at an earlier life stage despite exposure to potentially traumatic experiences. It is important to examine when, why, and whether these experiences may have caused a disbalance in the relationship between stress and resilience capacities and may have provoked mental health problems. Then, it is important to learn how they were previously dealt with, and if and how the balance was restored. Clinicians should identify the index trauma that has initially impacted survivors’ development, learn about the fluctuation of psychopathology and resilience potentials throughout a life trajectory, and a hierarchy of survivors’ needs while seeking help (18, 21).

Next, patients’ expectations from help should be identified and negotiated within a treatment plan. We inform patients that targeting the index trauma with a trauma-focused intervention may be the most recommendable path in healing their multiple psychological problems. We believe that working through intense emotions attached to the index trauma will improve patients’ self-regulatory emotional, social, and psychological capacities (18). However, some patients may disagree with this perspective. These may have unsuccessfully been treated for traumatic experiences earlier, and have suffered from a worsening of complaints thereupon. In some cases, a trauma-focused intervention was applied inadequately, not having the index trauma as a focus. In others, treatment was a short-term one or was started before a solid therapeutic alliance was established. Or was a therapist mindlessly following a treatment protocol without careful monitoring of the treatment process and overburdened a patient who was incapable of dealing with strong emotions at the time? Also, a patient may have had a different hierarchy of needs and was suffering more from interpersonal, attachment, and emotion regulation issues than from PTSD-related complaints. All these mentioned aspects should be taken into consideration and discussed with a patient first. The decision-making process should be a shared endeavor.

Personal history taking also enables clinicians to comprehend patients’ appraisals of critical life events, as appraisals determine whether life events will be experienced as traumatic or not (22). At the same time, it provides patients with an opportunity to make meaning of their life experiences, understand how they felt and behaved earlier in life, and why they have made certain life choices and not others. As asserted earlier, introspection is always retrospection, and history-taking is history-making (20). A cogent explanation, attuned to the survivor’s personal-developmental context, may offer relief by making sense of previously inexplicable feelings; it enables a sense of control (20).

Examining personal history on a content level helps build a therapeutic alliance with a trauma survivor. Throughout several initial hourly sessions, this alliance receives its foundation blocks. Inquiring into personal history in a curious, respectful, and humble way, creating thereby an opportunity for survivors to express feelings and share memories, helps establish trust in a therapeutic relationship. Safety, transparency, confidentiality, genuineness, positive unconditional regard, and spontaneity are the other essential ingredients that must be established. Patients should experience disclosure as a vehicle for healing psychological wounds, and clinician needs to develop accurate empathy and learn to see the world as a patient sees it. Research suggests that therapeutic alliance is the most critical ingredient of healing encounters and the best predictor of treatment success (23), more so than a therapeutic approach applied.

The Contextual Developmental Assessment interview (CDA) (24) may help with the task of collecting relevant anamnestic information in a structured manner and can be combined with the cross-culturally validated self-report International Trauma Questionnaire (ITQ) (14).

Moreover, the assessment phase should also include psychoeducation about C-PTSD, and inquire into co-morbid conditions, like dissociative, depressive, anxiety, and somatic symptom disorders, substance abuse, and physical health problems.

Careful personal history-taking is like putting a puzzle of one’s life together, eventually leading to a case conceptualization. Based on this conceptualization, three different groups of patients can be distinguished: those with C-PTSD due to early childhood trauma, those with C-PTSD due to trauma in adulthood, and a group presenting with a combination of both.

## Where to start the treatment: in or above the soil?

In the treatment of wounded trees, one can start intervening on a tree’s roots, in the soil, on a trunk and branches, or in a tree’s ecological environment above the soil. In cases where roots pose a threat to a tree’s growth, these should be eliminated, and exposed roots should be carefully trimmed by hand. Thereby, the soil should be dampened, and then the soil’s top layer should be gently loosened. Also, a tree may need more sunlight or minerals must be added to reduce soil’s acidity. Proper pruning practices apply to the branches as well. Dried ones need to be carefully pruned by hand and crossing branches should be eliminated.

In the C-PTSD treatment, intervening on roots and in the soil is an analogy for targeting the impacts of childhood developmental trauma, while interventions above the soil apply to healing the impacts of traumatic experiences in adulthood, and those in childhood extending into adult age. All interventions can be combined and different approaches can thereby be applied.

**Figure 2:**
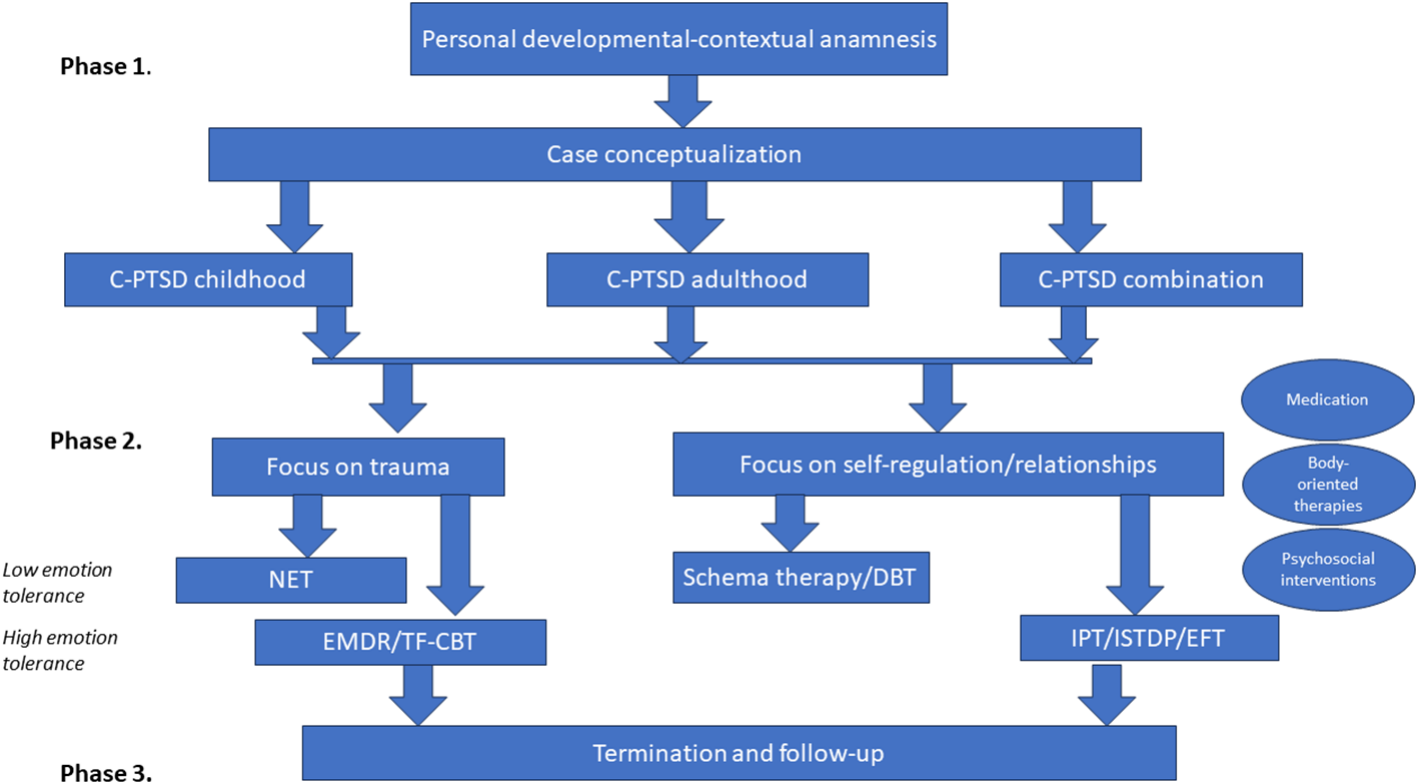
The C-PTSD treatment strategy.

The C-PTSD treatment can start either with trauma-focused therapy or with therapy targeting interpersonal and identity issues. The choice depends on patients’ needs and preferences and clinical judgment and should be negotiated. Sequencing and timing of treatment interventions need to be carefully carved, considering patients’ abilities to tolerate strong emotions. This capacity is determined by patients’ unique psychological gestalt, and current level of stress in their ecological environments (**Figure 2**). We usually recommend applying both, interventions aiming at fear extinction and those having cognitive restructuring as the main target.

In the cases where patients prioritize interpersonal and, or identity problems, and have limited capacity to tolerate strong emotions, healing may proceed with schema therapy (25). This is an integrative psychotherapy combining theory and techniques from cognitive behavioral therapy, psychoanalytic object relations theory, attachment theory, and Gestalt therapy. It aims at breaking ingrained patterns that individuals develop through their early psychosocial development and which become dysfunctional later on in life. Schema therapy was originally designed for the treatment of personality and depressive disorders, both common co-morbid conditions to C-PTSD. It can be applied in a structured and manualized way. An alternative to schema therapy may be dialectical behavioral therapy (DBT), also originally developed for the treatment of personality disorders (26).

In the cases where patients can tolerate intense emotions, and have a well-developed capacity for psychological insight, we suggest applying Emotion-focused therapy (EFT), Interpersonal therapy (IPT), or Intensive short-term dynamic psychotherapy (ISTDP). EFT (27), is based on the premise that emotions are crucial to identity and that they guide individual choice and decision-making. This type of therapy assumes that lacking emotional awareness or avoiding unpleasant emotions can cause harm. IPT and ISTDP also aim at understanding emotions and targeting defense mechanisms. These three approaches focus more on the process, on the here-and-now unfolding in therapeutic encounters, rather than on the content. A therapeutic relationship is the principal agent of change, as all patients’ interpersonal problems may emerge within a healing encounter. Scientific evidence regarding the effectiveness of these three approaches in C-PTSD treatment is promising, but still in its early stage (28–32).

While healing a wounded tree, one should avoid over-mulching and overwatering of dry soil, and provide an optimal amount of moisture and nutrients. Also, it should be kept in mind that young trees need more water than mature ones. We use this analogy to explain the concept of limited reparenting which has a valuable role in all approaches for the treatment of interpersonal and identity impacts of developmental trauma mentioned.

Limited reparenting aims at meeting patients’ needs that were previously unmet in their childhood. It helps patients to identify experiences that were missed and create corrective experiences within a safe therapeutic relationship. It is believed that these corrective experiences serve as an antidote to damaging experiences that have led to the development of maladaptive interpersonal interactions.

When patients consider PTSD complaints as their main problem, we suggest applying one of the evidence-based trauma-focused therapies. The choice for a particular approach depends again on patients’ ability to tolerate strong emotions. We recommend using NET where exposure to high levels of anxiety in a course of treatment may cause psychotic and dissociative phenomena, or worsening of patients’ condition otherwise. EMDR and TF-CBT should be used when patients can withstand strong emotions. In patients treated with EMDR and TF-CBT and presenting with a combination of early childhood with adulthood traumas, an agreement should be made regarding which traumatic experience should be targeted first. Treatment may start with the index childhood trauma and proceed onwards with later traumatic experiences. Alternatively, it may target the most recent trauma and proceed backward, or start with the most impacting traumatic experience. We believe that the first and the third options are the recommended ones. However, the rationale for the second one is that by targeting the most recent traumatic experience patients’ allostatic load will be reduced. This will make working through earlier traumatic experiences less emotionally overwhelming.

If necessary, EMDR, TF-CBT, and NET can be followed by schema therapy, DBT, EFT, IPT, or ISTDP, and vice versa. This may be needed when PTSD complaints continue causing significant suffering upon resolving interpersonal and, or identity issues, or when C-PTSD problems become more prominent upon (partial) remission of PTSD complaints. The two approaches can also be used intermittently throughout a treatment course. When interventions targeting interpersonal and identity issues provoke high anxiety levels in patients and throw them out of the optimal arousal zone (the “window of tolerance”) (33), EMDR and TF-CBT sessions can be added. These aim at lowering or extinguishing fear and at changing incompatible depressive cognitions. EMDR can also be added when anger and rage are patients’ dominant emotions. The EMDR anger and rage protocol can be of great use here.

To conclude, we suggest that effective C-PTSD treatment is an alternating sequence of affect evocation and analysis followed by affect integration (20). Survivor’s emotional expression should be encouraged throughout treatment, and emotions should be analyzed and understood from the personal developmental-contextual perspective. Herewith, affect expression, establishment of corrective experiences in a healing relationship, and meaning-making will gradually occur. Clinicians should switch focus from content to process and back throughout a treatment course while combining trauma-focused interventions with those focusing on emotional self-regulation and relational issues.

Last but not least, trees need attention and time to heal. We suggest that effective C-PTSD treatment is also a time-consuming endeavor, and a process in need of careful monitoring and critical evaluation throughout.

## Additional remedies

Overall, we suggest combining both approaches described with those targeting patients’ coping and resilience capacities. It is helpful to consider what enabled patients to cope with posttraumatic impacts and other adversities earlier in life, and whether some of the previously applied strategies may be successfully re-used. Moreover, clinicians should examine which new coping strategies patients can develop, and how they may improve the use of resources in their environments. Resilience-focused interventions may precede other interventions in cases where patients’ ability to tolerate strong emotions seems to be weak, and the level of ongoing stress is high. They can also follow them when the treatment effect is unsatisfactory, or alternate with them at times when intense emotions seem to be flooding a patient. Trimming of branches to stimulate a tree’s growth may be an analogy for resilience-oriented interventions.

In cases where patients are confronted with ongoing stressful life events (ex. asylum seekers facing existential uncertainty; refugees confronted with ongoing war in their countries of origin, and with uncertainty about the whereabouts of those left behind; ongoing family and marital abuse or financial problems), using the suggested approaches only may not be enough to achieve lowering of suffering and impairment in patients’ daily life functioning. We recommend preceding and, or combining the suggested approaches with psychosocial interventions to diminish the impact of ongoing stress. These include collaboration with legal personnel, social workers, activity centers, additional family or marital therapy sessions, short-term inpatient treatment, etc. Where necessary and possible, treatment interventions should be directed not only at the output (impacts of traumatic experiences) but also at the input (ongoing psychosocial adversities) of stress. Referring to the tree metaphor, a scaffolding should sometimes be constructed around a tree’s trunk to prevent it from inclining while being subjected to transient environmental stress.

In some C-PTSD patients, psychotherapy should be combined with the administration of psychotropic medication. Psychopharmacological treatment is sometimes a useful vehicle for making psychotherapy feasible and, or for accelerating psychotherapy progress. The medication targets pervasive and invalidating PTSD and co-morbidity symptoms. It also helps patients to stay within optimal arousal zone (33) and enhances tolerance for intense emotions.

However, psychotropics should ideally not be applied extensively and for an extended time. We recommend clinicians to be guided by the “not too little, not too much, and as time-limited as possible” principle. More on the use of specific psychopharmacological agents can be found elsewhere (14, 34). Referring again to the tree metaphor, the use of weed fertilizers and pesticides in healing should be minimized and controlled, as these contain toxins that can kill beneficial organisms and compromise a tree’s resilience. A tree may, in the long run, also become dependent on fertilizers.

Therapy-accelerating devices (20) are valuable tools in C-PTSD treatment. They can be applied to “unfreeze” patients, help them cope with emotional ambivalence, and generate data for subsequent exploration. Patients may be suggested to write letters to those they have unfinished business with and discuss them with a clinician. This assignment consists of writing one letter about how patients have experienced being harmed by another person, another one about how they have experienced all the good things that that person did to them, and a third one merging both perspectives and attached emotions. Or patients may be asked to write down comforting thoughts, collect comforting photos or other objects and create a “box for support”. They can use this box in between therapy sessions as a transitional object at times when they feel tormented. Home visits, bringing old family photos to a session, or the empty-chair technique are other recommendable therapy-accelerating means.

Last but not least, psychotherapy can be combined with treatment approaches using the body (rather than cognition or emotion) as a primary entry point in processing trauma. Unassimilated somatic responses evoked in trauma, involving both arousal and defensive responses, contribute to many C-PTSD symptoms. Sensorimotor psychotherapy (35) directly treats the effects of trauma on the body, which in turn facilitates emotional and cognitive processing.

## When will a tree become healthy (enough) again?: treatment duration

Trees need a long time to heal, up to 15 or 20 years. Unlike humans, they cannot repair or replace damaged tissue. Instead, they attempt to “seal” it off from the healthy one. Psychological wounds of adult psycho-trauma survivors can be both “sealed off”, leaving a scar behind, and repaired.

Keeping in mind the chronic nature of C-PTSD, complete remission of symptoms is often an unreachable treatment goal. Instead, we suggest that the treatment should aim at re-establishing the balance between psychopathology and resilience potentials to the extent that survivors’ daily functioning and quality of life are improved despite the presence of subthreshold PTSD symptoms (18). This goal appears to be more realistic and should be communicated transparently with patients. Agreeing upon a reachable treatment goal is essential for managing the duration of the healing process. Also, it enables treatment termination without disappointment regarding achieved results. Referring to and eventually reframing and re-negotiating initial treatment goals and expectations can be helpful while discussing treatment termination with patients. Moreover, we argue that discussing patients’ meaningful future life goals is very important. Some survivors may become depressed and disoriented once their long-time struggle with C-PTSD complaints diminishes or comes to an end.

We propose that the availability of a holding treatment environment for a longer term (admittedly with a scaled-down frequency of sessions) is vital for increasing the sustainability of the treatment effects. However, with long-term contact, the risks of overprotecting patients and making them dependent on a therapeutic relationship should be closely monitored. Safety and predictability of a holding treatment environment are not treatment goals per se, but vehicles for re-establishing patients’ internalized feelings of psychological safety. When established, patients are expected to be able to extrapolate these feelings to the outside world, beyond a treatment setting.

We suggest that C-PTSD treatment should last “as short as possible and as long as there is progress present,” meaning that it should be terminated when patients do not show signs of psychological change and growth anymore and when clinicians notice that therapeutic interventions used are ceasing to sort effect. Therefore, the treatment process should be evaluated regularly. Long-term supportive contact should be offered by social workers, other professionals, and non-professionals.

In our experience, the C-PTSD treatment described in this article can be completed in about 2 to 3 years on average, provided that circumstances in patients’ ecological environment are relatively favorable and stable. In the beginning, treatment sessions take place on a weekly or bi-weekly basis, and frequency is reduced in the termination phase to once a month or less for more extended time. However, this is an approximation of the treatment duration, as patients are individuals with unique needs and life circumstances, and we aim to provide matched care.

## Discussion

The C-PTSD treatment strategy proposed in this article is a blend of trauma-focused treatments and psychotherapies aiming at improving patients’ emotional self-regulation and relational capacities. It has more in common with the sequenced than the unimodal approach and it is in line with the most recently recommended C-PTSD treatment strategies (14). We consider case conceptualization paramount to our strategy. Clinicians are advised not to rush with establishing a diagnosis and proceeding with a treatment protocol as soon as possible. They should first understand the underlying dynamics of processes leading to psychopathology and build a safe therapeutic alliance. The importance of adequate assessment of the effects of traumatic experiences on a survivor and his/her ecological environment has also been recently stressed elsewhere (14).

Next, we make a clear distinction between different types of C-PTSD patients in need of different types of treatment. In our approach, patients experiencing emotional self-regulation and relational challenges as their main problems are targeted with evidence-based psychotherapies originally developed for the treatment of personality disorders. The sequenced approach also incorporates schema therapy elements, but only in the stabilization phase. According to the manual, this is limited to 4 sessions and aims to provide a framework for psychoeducation (36). In our approach stabilization occurs throughout the process of personal history-taking and building of a therapeutic alliance, while schema therapy is a separate treatment component. Moreover, patients can in the stabilization phase be additionally supported with psychosocial interventions and medication upon indication.

The other difference between the suggested approach and the sequenced treatment (36) is type of the trauma-focused therapy used in patients who consider PTSD-related complaints as their main problem. While NET is used in the second phase of the sequenced approach, we suggest using it specifically when patients have low tolerance for strong emotions. In other patients, we recommend using EMDR and TF-CBT. Although these two approaches have been found equally effective in PTSD patients (5), we suggest applying both in the C-PTSD treatment.

While the unimodal approach with EMDR was most often successfully used in populations with relatively circumscribed traumatic experiences (37), we suggest applying this treatment technique in complex posttraumatic presentations. Like in the unimodal approach, we argue that the effects of trauma-focused therapy may ripple beyond PTSD complaints and can improve, to a certain extent, emotional self-regulation, relational capacities, and other co-morbidity in C-PTSD patients. Moreover, we argue that the same applies the other way around and that schema therapy, DBT, EFT, IPT, and ISTDP can diminish PTSD and other co-morbidity symptoms on their turn while having emotional self-regulation and improvement of relational capacities as main targets. This occurs through the lowering of fear and re-establishing a sense of agency and control in patients.

Finally, the approach described lasts longer than the unimodal one and is similar to the sequenced one with an equal amount of sessions spread over a longer period.

Yet, the model proposed in this article still lacks sound scientific support, and its acceptability and effectiveness need to be studied. The suggested interventions should be investigated as standalone treatments for C-PTSD and as components within the multicomponent treatment program. These studies should include individuals formally diagnosed with C-PTSD (14). The optimal duration of the proposed approach has to be established, too. Assessment of a wide range of outcomes, including amelioration of symptoms, enhancement of resiliency, and improvement of functional impairment, needs to be determined. The dynamic of change between different symptom clusters of C-PTSD throughout the treatment should be investigated to guide the flexibility needed in sequencing the interventions.

Finally, psychotherapy of C-PTSD can profit from advances in neurobiology research. Differences between PTSD and C-PTSD are not sufficiently established yet, and more studies focusing on the effect of trauma type on brain maturation are needed (14). Neuroendocrine research on C-PTSD is currently unavailable and should be developed. Although pharmacological treatment of C-PTSD is not recommended as a standalone intervention (14), novel approaches in the treatment of PTSD, like MDMA (38) or ketamine (39), may open new avenues in healing complex posttraumatic sequelae.

## Conclusion

The treatment approach presented is based on longstanding clinical observations and experience, as well as recent theoretical considerations. It may be a promising framework for C-PTSD treatment. C-PTSD treatment should not be a Procrustean bed, arbitrarily forcing patients to fit into rigid patterns of treatment protocols. Patients may better be approached in a personalized way, considering their unique personal psychosocial developments, needs, and expectations. We encourage clinicians to be curious, ready to sacrifice the certainty that accompanies orthodoxy, and to apply this approach in daily practice.

However, this approach needs to be subjugated to scientific scrutiny. Therefore, we invite researchers to examine its acceptability and effectiveness. With this, taking both PTSD symptoms and other areas of psychosocial functioning as outcome measures, and inquiring into its long-term effects is crucial.

## Data availability statement

The original contributions presented in the study are included in the article/Supplementary Material. Further inquiries can be directed to the corresponding author.

## Author contributions

BD: Writing – original draft, Writing – review & editing. JR: Writing – original draft, Writing – review & editing.
